# CD73^+^ Dendritic Cells in Cascading Th17 Responses of Experimental Autoimmune Uveitis-Induced Mice

**DOI:** 10.3389/fimmu.2020.601272

**Published:** 2020-12-02

**Authors:** MinHee K. Ko, Hui Shao, Henry J. Kaplan, Deming Sun

**Affiliations:** ^1^ Doheny Eye Institute and Department of Ophthalmology, David Geffen School of Medicine at UCLA, Los Angeles, CA, United States; ^2^ Department of Ophthalmology and Visual Sciences, Kentucky Lions Eye Center, University of Louisville, Louisville, KY, United States; ^3^ Department of Ophthalmology, Saint Louis University (SLU) Eye Institute, SLU School of Medicine, Saint Louis, MO, United States

**Keywords:** autoimmunity, adenosine receptors, bone marrow dendritic cells, bone marrow culture dendritic cells, CD73, experimental autoimmune uveitis, γδ T cells, uveitis

## Abstract

Previous studies have shown that CD73 is pivotal in the conversion of pro-inflammatory adenosine triphosphate into anti-inflammatory adenosine and that immune cells of the same type that express different levels of CD73 are functionally distinct. In this study we show that adenosine enhances the Th17 promoting effect of dendritic cells (DCs), and DCs expressing CD73 critically augment Th17 responses. Bone marrow dendritic cells (BMDCs) do not constantly express CD73; however, a significant portion of the BMDCs expressed CD73 after exposure to Toll-like receptor ligand, leading to stronger Th17 responses by converting adenosine monophosphate to adenosine. We show that the CD73^+^ BMDCs play a critical role in cascading Th17 responses, and CD73^+^ BMDCs are functionally augmented after treatment with Toll-like receptor ligand. Splenic antigen presenting cells (DCs) of CD73^−/−^ mouse have a poor Th17-stimulating effect, even after exposure to lipopolysaccharide (LPS) or γδ T cells, indicating that induction of CD73^+^ DCs is critically involved in augmented Th17 responses. We conclude that CD73^+^ DCs critically trigger cascading Th17 responses, and the activated Th17 cells that express CD73 further augment Th17 responses, leading to cascading exacerbation. Hence, disabling the CD73 function of DCs should block this cascading response and mitigate Th17 responses.

## Introduction

Under pathologic conditions, a large amount of adenosine triphosphate (ATP) is released into the extracellular compartment by injured and dying cells (1–4). The released ATP acts as an endogenous Toll-like receptor (TLR) ligand to enhance immune responses and inflammation (5–9). A combination of the ectoenzymes CD39 and CD73 degrades ATP, adenosine diphosphate (ADP), and adenosine monophosphate (AMP) to adenosine, thereby quenching the ATP-driven proinflammatory processes (10). Consequently, generated adenosine counteracts ATP-mediated immune stimulation, preventing uncontrolled inflammation and lessening the collateral damage to healthy tissues (1, 2, 11). Hence, the metabolic process of ATP conversion to adenosine has been viewed as an ‘immunological switch’ that shifts ATP-driven proinflammatory immune cell activity toward an anti-inflammatory state (12–14).

The glycosylphosphatidylinositol-linked membrane protein CD73 is the main enzyme responsible for the conversion of AMP into immunosuppressive adenosine (12, 13, 15–17). Studies have shown that CD73-deficient mice have altered inflammatory reactions (18–20). Expression of CD73 is functionally important for Foxp3^+^ regulatory T cells (10, 21), Myeloid-derived suppressor cells (22), and M2 macrophages (23–25), as well as for γδ T cells (26, 27); and immune cells of the same type that express different levels of CD73 are functionally distinct (24, 25). To determine whether dendritic cells (DCs) express CD73 and whether CD73 is functionally important for DCs, we examined CD73^+^ cells among cultured bone marrow dendritic cells (BMDCs) and splenic CD11c^+^ cells.

DCs are the principal antigen-presenting (AP) cells for initiating immune responses. Previous studies showed that DC differentiation and function is profoundly affected by TLR ligand (28) and adenosine (23, 29, 30). Since levels of extracellular adenosine increase greatly during inflammation (31–33), and since our previous studies showed that adenosine has an opposite effect on Th1 and Th17 pathogenic responses in experimental autoimmune uveitis, we examined whether adenosine and adenosine metabolism of DCs contribute to a biased effect of adenosine on Th1 and Th17 responses: particularly, whether CD73-expressing DCs differ in supporting Th1 vs Th17 responses. Our results show that BMDCs cultured with granulocyte–macrophage colony-stimulating factor (GM-CSF) do not express detectable levels of CD73; however, a significant portion of the BMDCs become CD73^+^ after exposure to TLR ligand or γδ T cells, suggesting that CD73 is not constantly expressed by BMDCs but is inducible. Functional comparison between induced CD73^+^ and CD73^-^ BMDCs and between BMDCs from CD73^+/+^ (wt-B6 mouse) and CD73^−/−^ (CD73^−/−^ mouse) showed that CD73^+^ BMDCs are stronger stimulators for IL-17^+^ T cells whereas CD73^−^ BMDCs preferentially stimulate Th1 responses. The CD73^+^ BMDCs produce unique patterns and amounts of cytokines as compared to CD73^−^ DCs. Our results demonstrated that the induction of CD73^+^ DCs is crucially involved in cascading Th17 responses and that disabling CD73 function on DCs effectively mitigates the Th17 pathogenic responses in autoimmune diseases.

## Materials and Methods

### Animals and Reagents

Female C57BL/6 (B6) mice and CD73^−/−^ and TCR-δ^−/−^ mice were purchased from Jackson Laboratory (Bar Harbor, ME); 12- to 16-week-old mice were used in all studies. All mice were housed and maintained in the animal facilities of the University of California Los Angeles. Institutional approval (Protocol number: ARC#2014-029-03A) was obtained from the Institutional Animal Care and Use Committee of the Doheny Eye Institute, University of California Los Angeles, and institutional guidelines regarding animal experimentation were followed. Veterinary care was provided by IACUC faculty. Immunized animals that displayed swelling joints were either humanely euthanatized or administered an analgesic (buprenorphine, 0.1 mg/kg sc. twice daily or ketoprofen, 2 mg/kg sc. daily) until the swelling resolved. By the end of the study, mice were euthanized by cervical dislocation after a lethal injection of ketamine and xylazine prior to tissue collection. Recombinant murine IL-1β, IL-7, IL-12 and IL-23 were purchased from R & D (Minneapolis, MN). Fluorescein isothiocyanate (FITC)-, phycoerythrin (PE)-, or allophycocyanin (APC)-conjugated antibodies (Abs) against mouse CD4 (GK1.5), αβ T cell receptor (TCR) (H57-597), or γδ TCR (GL3) and their isotype control antibodies were purchased from Biolegend (San Diego, CA). (PE)-conjugated anti-mouse IFN-γ (XMG1.2) and IL-17 (TC11-18H10.1) monoclonal antibody was purchased from Santa Cruz Biotechnology (Dallas, Texas). The non-selective AR agonist 5’-N-ethylcarboxamidoadenosine (NECA) (34) and AMP were purchased from Sigma-Aldrich (St. Louis, MO, USA). Toll-like receptors ligand LPS was purchased from Invivogen (San Diego, CA).

### Immunization and EAU Induction

EAU was induced in B6 mice by subcutaneous injection of 200 μl of emulsion containing 200 μg of human IRBP_1-20_ (Sigma-Aldrich, St. Louis, MO) in complete Freund’s adjuvant (CFA; Difco, Detroit) at six spots at the tail base and on the flank and intraperitoneal (i.p.) injection with 300 ng of pertussis toxin.

### T Cell Preparation

All αβ T cells used were purified from the spleen or draining lymph nodes of IRBP_1–20_-immunized mice at day 13 post-immunization using an auto-MACS separator system, as described previously [29]. The purity of the purified cells was >95%, as determined by flow cytometric analysis using phycoerythrin-conjugated antibodies against αβ T cells. The cells were then cultured in RPMI 1640 medium containing 10% fetal calf serum (Corning).

### Prepare γδ T Cells

Non-activated and activated γδ T cells were separated from either naïve B6 mice or IRBP_1–20_-immunized B6 mice, respectively, by positive selection using a combination of FITC-conjugated anti-TCR-δ antibody and anti-FITC antibody-coated Microbeads, followed by separation using an auto-MACS.

### Generation of Bone Marrow Dendritic Cells

Bone marrow dendritic cells were generated by incubating bone marrow cells for 5 days in the presence of 10 ng/ml of recombinant murine GM-CSF and IL-4 (R&D Systems), as described previously (35). Cytokine (IL-1β, IL-6, L-12 and IL-23) levels in the culture medium were measured by ELISA.

To determine the antigen-presenting function, BMDCs were incubated in a 24-well plate with responder T cells isolated from immunized B6 mice under Th1- or Th17-polarizing conditions. Forty-eight hours after stimulation, IFN-γ and IL-17 in the culture medium were measured by ELISA. The percentage of IFN-γ^+^ and IL-17^+^ T cells among the responder T cells was determined by intracellular staining after 5 days of culture as described above.

### Adenosine Assay

Adenosine in the medium of cultured cells was measured by an Adenosine Assay Kit (Fluorometric) from Biovision (CA). Briefly, 25 µl of cultured cell supernatant were mixed with assay buffer, adenosine convertor, adenosine detector, adenosine developer and adenosine probe from the kits to compose a 100 µl reaction system. Kept in room temperature for 15 min and protected from the light. Fluorescence was read in a SpectraMax iD5 multi-mode microplate reader (Molecular Devices, LLC. USA) at Ex/Em = 535/587 nm.

### CFSE Assay

Purified CD3^+^ T cells from IRBP_1–20_-immunized TCR-δ^−/−^ mice were stained with CFSE (Sigma-Aldrich) as described previously (36). Briefly, the cells were washed and suspended at 5 × 10^6^ cells/ml in serum-free RPMI 1640 medium; cells were then incubated at 37°C for 10 min with gentle shaking with a final concentration of 5 μM CFSE before being washed twice with, and suspended in, complete medium, stimulated with anti-CD3 antibodies which were pre-coated on 24 well plate. Some 48 h later, the T cells were harvested and analyzed by flow cytometry.

### Experimental Setting for Interaction of T Cells and Dendritic Cells and Measurement of Th1 and Th17 Responses

In a 6-well plate, 2 × 10^6^/well BMDCs were co-cultured with 1 × 10^5^/well γδ or αβ T cells for 24 h. After DCs were separated from T cells, the BMDCs were irradiated (5,000 Rad) and seeded in a 24-well plate at 3 × 10^4^/well with CD3^+^ cells isolated from immunized B6 mice. Five days later, the percentage of IFN-γ^+^ and IL-17^+^ T cells among the responder T cells was determined by intracellular staining, followed by FACS analysis, as described previously (37).

αβ T cells (1.8 × 10^6^) were collected from IRBP_1-20_-immunized B6 mice on day 13 post-immunization. To obtain enough cells, the cells obtained from all six mice in the same group are routinely pooled before the T cells are further enriched. The cells were co-cultured for 48 h with irradiated spleen cells (1.5 × 10^6^/well) as APCs and IRBP_1–20_ (10 μg/ml) in a 24-well plate under either Th1 (culture medium supplemented with 10 ng/ml of IL-12) or Th17 polarized conditions (culture medium supplemented with 10 ng/ml of IL-23) (37, 38). Cytokine (IFN-γ and IL-17) levels in the serum and 48 h of culture supernatants were measured by ELISA (R & D Systems).

### ELISA Measurement of Cytokine

ELISA kits (E-Bioscience) were used to measure serum IFN-γ and IL-17 levels on day 13 post-immunization and in the 48 h culture supernatants of responder T cells isolated on day 13 post-immunization from IRBP_1–20_-immunized B6 or TCR-δ^−/−^ mice.

### Statistical Analysis

The results in the figures are from a representative experiment, which was repeated 3–5 times. We used 2-way Students t-tests unless otherwise specified. Data were presented as the means with error bars for standard error of mean (SEM). Statistical analysis and graphing were performed in Excel software (Microsoft Corp). Asterisks (**) in graphs indicated p ≤0.05 representing statistical significance.

## Results

### Adenosine Augments DCs’ Th17 Promoting Activity

**Figure 1:**
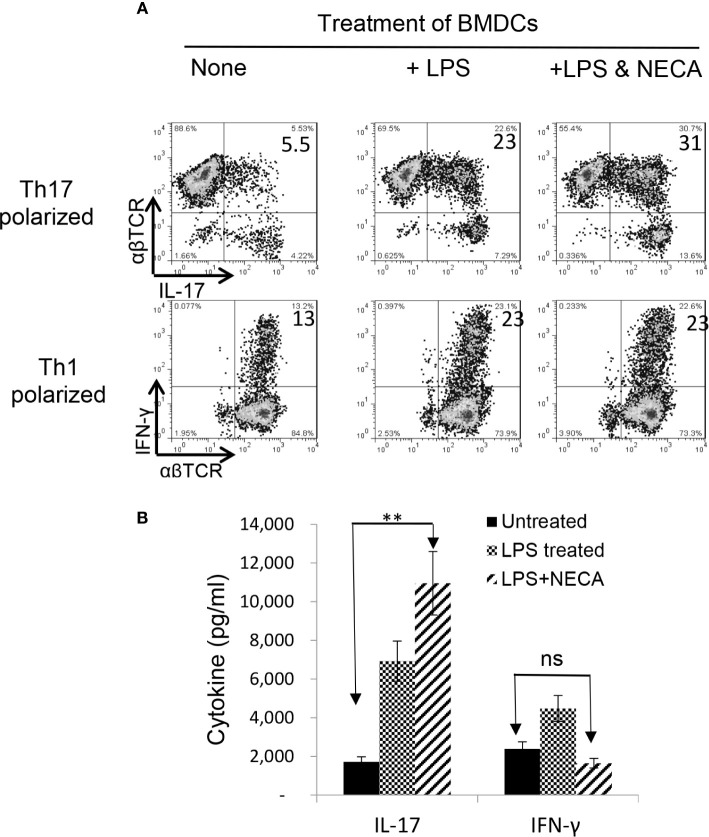
Adenosine augments DCs’ Th17 promoting activity **(A)**. Exposure of bone marrow dendritic cells (BMDCs) to a non-selective adenosine receptor agonist NECA enhanced DCs’ Th17-promoting activity. BMDCs were generated by incubation of bone marrow cells in the presence of granulocyte-macrophage colony-stimulating factor and IL-4 (35). They were treated or untreated with lipopolysaccharide (LPS, 100 ng/ml)) or LPS + NECA (100 nM). In 24 well plated, the αβ responder T cells obtained from immunized B6 mice were stimulated *in vitro* with the immunizing antigen and the treated BMDCs at ratio of DC:T = 1:20. The numbers of IL-17^+^ T cells activated after a 5-day *in vitro* stimulation under Th17 polarizing conditions were examined **(B)**. ELISA assay measuring IFN-γ and IL-17 production of αβTCR^+^ responder T cells. IL-17 production by responder T cells were also enhanced by NECA treatment, whereas the IFN-γ production was inhibited. The data are from one single experiment and are representative of those obtained in three independent experiments. Values are expressed as mean ± SEM (*n* = 6). **p < 0.05. ns, not significant.

Our recent studies showed that adenosine has a distinct effect on Th17 versus Th1 responses; it tips the balance between Th1 and Th17 responses towards the latter (38, 39). To determine the contribution of DCs to such contradictory effects of adenosine on antigen-specific Th1 and Th17 responses, we examined the antigen presenting (AP) function of BMDCs, before and after exposure to NECA—a non-selective adenosine receptor agonist (34). The αβTCR^+^ responder T cells obtained from immunized B6 mice were stimulated *in vitro* with the immunizing antigen and the treated BMDCs at ratio of DC:T = 1:20 under Th1 (culture medium added with IL-12) or Th-17 (culture medium added with IL-23) polarizing conditions (39, 40). Th17 responses were assessed by evaluating numbers of IL-17^+^ T cells activated after a 5-day *in vitro* stimulation under Th17 polarizing conditions. After a 5-day *in vitro* stimulation by the immunizing antigen, under Th1 or Th17 polarizing conditions (27, 38), a significantly increased number of the CD3^+^ responder T cells became IFN-γ and IL-17 positive after stimulation by lipopolysaccharide (LPS)-treated BMDCs (**Figure 1A**, upper panel). The IL-17^+^, but not the IFN-γ^+^, cells further increased if BMDCs were dually treated with LPS and NECA (**Figure 1A**, lower panel). Cytokine tests (**Figure 1B**) showed that IL-17 production by responder T cells was also enhanced after stimulation by BMDCs dually treated with LPS and NECA, whereas the IFN-γ production was inhibited.

### LPS Treatment Triggers BMDCs’ Response to AMP

**Figure 2:**
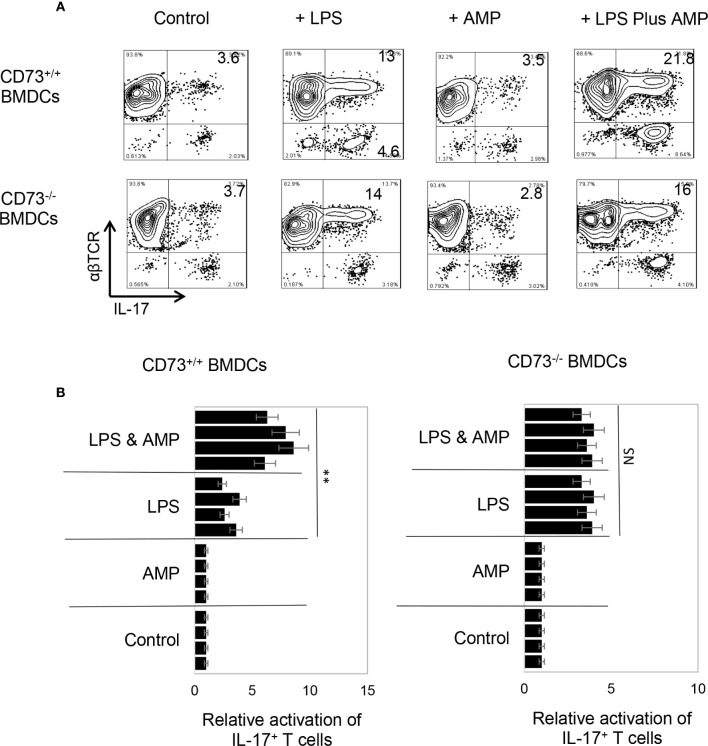
Bone marrow dendritic cells acquire response to adenosine monophosphate after lipopolysaccharide treatment **(A)**. Synergistic effect between lipopolysaccharide (LPS) and adenosine monophosphate (AMP) in enhancing bone marrow dendritic cells’ (BMDCs’) Th17 promoting activity. BMDCs from B6 (CD73^+/+^) (upper panels) or CD73^−/−^ (lower panels) mouse were examined for antigen presenting function, after exposure to LPS (100 ng/ml) or LPS plus AMP (1 μM). Responder T cells obtained from immunized B6 mice. The numbers of IL-17^+^ T cells among responder T cells were examined after staining with anti-IL-17 and anti-αβTCR mABs, after a 5-day *in vitro* stimulation under Th17 polarizing conditions. The data are from one single experiment and are representative of those obtained in three independent experiments **(B)**. Summarized data of four separate experiments showing that AMP enhanced BMDCs’ Th17 stimulating effect only after LPS treatment (left panels) and that the CD73^−/−^ BMDCs remained unresponsive to AMP even LPS treatment (right panels). Values of relative activation of Th17^+^ T cells were calculated by number of IL-17^+^ T cells in testing group divided by number of IL-17^+^ T cells in the control group (in the absence of LPS and AMP) and are expressed as mean ± SEM (n = 4). **p < 0.05. ns, not significant.

To determine whether AMP has a similar effect on BMDCs or whether BMDCs can convert AMP to adenosine and thus augment Th17 responses, the AP function of BMDCs was examined, before and after treatment with AMP, with or without a prior LPS treatment. Results in **Figure 2A** (upper panels) showed that AMP enhances Th17 promoting activity in LPS-treated, but not untreated, BMDCs. The number of IL-17^+^ T cells among the responder T cells was higher when BMDCs were pretreated with LPS. The number was further increased when the LPS-treated BMDCs were additionally treated with AMP (**Figure 2B**). However, BMDCs that were not treated with LPS did not respond to AMP, suggesting that LPS-treatment enabled BMDCs to respond to AMP. The effective doses of AMP range are from 1 to 8 µM and dose dependent effect was not apparent. Therefore, in subsequent studies we used the concentration of 1 µM.

### AMP Augmented Th17 Response but Only in the Presence of CD73^+/+^, but Not CD73^−/−^ BMDCs

**Figure 3:**
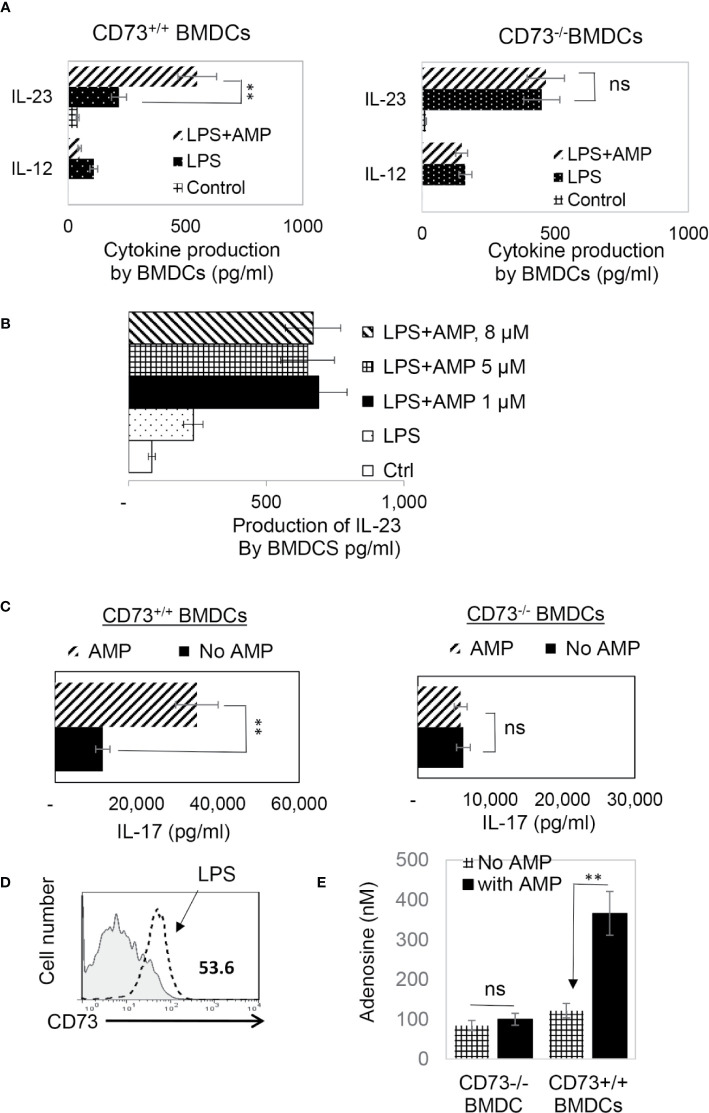
In response to AMP LPS-treated CD73^+/+^, but not CD73^−/−^, BMDCs produced greater amounts of IL-23 and acquired greater Th17 stimulating activity **(A)**. ELISA assays for IL-12 and IL-23 production by CD73^+/+^ and CD73^−/−^ BMDCs. 48h stimulated culture supernatants of CD73^+/+^ and CD73^−/−^ BMDCs were assessed for IL-12 and IL-23, after being stimulated with LPS or LPS plus AMP. The control BMDCs remained untreated. The data are from one single experiment and are representative of those obtained in three independent experiments. Values are expressed as mean ± SEM (*n* = 6). **p <0.05. ns, not significant **(B)**. ELISA assay test AMP doses ranging from 1 to 8 µM showed no apparent dose dependent effect. The experimental setting was the same as **Figure 3A** **(C)**. ELISA assays for IL-17 production by responder T cells after 48h stimulation by the immunizing antigen and CD73^+/+^ or CD73^−/−^ BMDCs. The data are from one single experiment and are representative of those obtained in three independent experiments **(D)**. LPS exposed BMDCs expressed increased amount of CD73. BMDCs were treated (dotted line) or untreated with a small dose of LPS (50 ng/ml) before stained with anti-CD73 mAB followed by FACS analysis **(E)**. Adenosine assay. Adenosine in the supernatants of cultured BMDCs was measured by an Adenosine Assay Kit (Fluorometric) after the CD73^+/+^ or CD73^−/−^ BMDCs were treated with LPS (100 ng/ml) with or with additional treatment of AMP (1 μM). ns, not significant.

CD73 is the main ecto-enzyme converting AMP to adenosine (12, 15). To test the prediction that LPS treatment enables BMDCs to express CD73, which allows BMDCs to convert AMP to adenosine, leading to augmented Th17 responses, we prepared CD73^+/+^ (isolated from CD73^+/+^ B6 mouse) and CD73^−/−^ BMDCs (isolated from CD73^−/−^ mouse) and compared the AP function between CD73^+/+^ and CD73^−/−^ BMDCs, before and after treatment with LPS (100 ng/ml) and/or AMP (1 μM) (**Figure 2A**). The results show that treatment with LPS enhanced the Th17-promoting effect of both CD73^+/+^ and CD73^−/−^ BMDCs; however, additional treatment of BMDCs with AMP augmented the Th17 responses presented by CD73^+/+^, but not by CD73^−/−^, BMDCs (**Figure 2A**, lower panels). These results indicate that CD73^−/−^ BMDCs were unresponsive to AMP and that CD73 expressed by BMDCs may convert AMP to adenosine, which augments Th17 responses (41). We also measured IL-12/IL-23 production of BMDCs, after treatment with LPS and/or AMP. As demonstrated in **Figures 3A, B**, LPS exposure induced both CD73^+/+^ and CD73^−/−^ BMDCs to produce increased amounts of IL-12 and IL-23. However, double exposure to LPS plus AMP enabled CD73^+/+^, but not CD73^−/−^, BMDCs to further increase IL-23 production and decrease IL-12 production.

Testing of AMP function by assessing cytokine production of responder T cells showed that AMP treatment further enhanced Th17 responses presented by CD73^+/+^ but not CD73^−/−^ BMDCs (**Figure 3C**). Phenotypic examination of BMDCs, before and after LPS treatment, showed (**Figure 3D**). LPS-treated BMDCs expressed significant levels of CD73, whereas the CD73 level had been undetectable before treatment. We also measured adenosine amounts in the cultured supernatants of CD73^+/+^ and CD73^−/−^ BMDCs after stimulation with LPS with or without AMP treatment. The results show that the adenosine concentration was significantly increased in AMP-treated CD73^+/+^ but not CD73^−/−^ BMDCs (**Figure 3E**).

### AMP Enhances Th17 Responses Supported by CD73^+/+^, but Not CD73^−/−^, Splenic DCs

**Figure 4:**
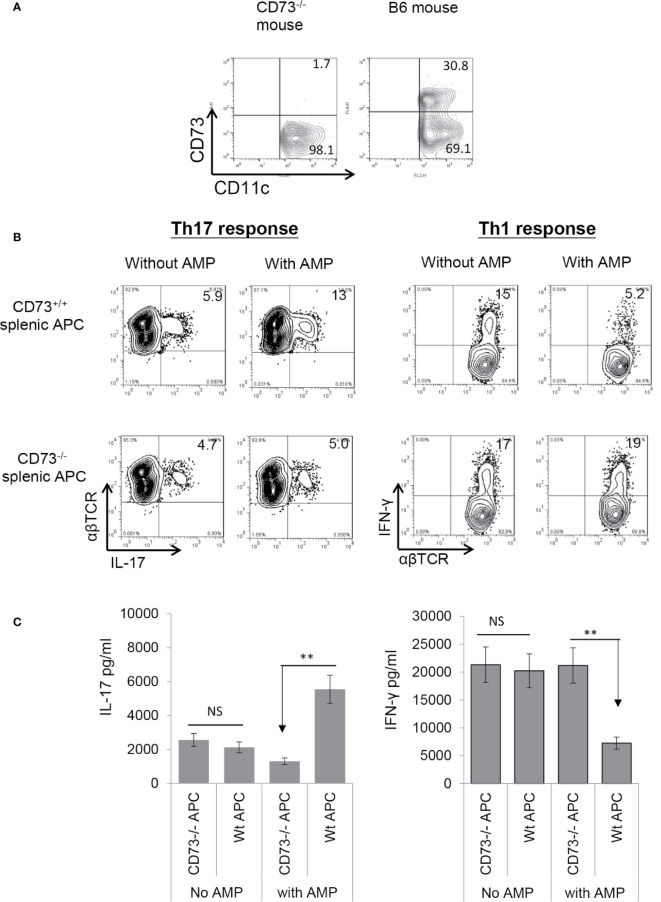
Adenosine monophosphate enhanced Th17 responses and inhibited Th1 responses in the presence of CD73^+^ splenic APCs **(A)**. Approximated one third of the splenic CD11c^+^ cells of B6, but not CD73^−/−^ mouse expressed CD73. Splenic cells of B6 and CD73^−/−^ mouse were double stained by FITC-anti-CD11c and PE-anti-mouse CD73. The staining profile of gated CD11c^+^ cells is shown **(B)**. MACS column separated αβ T cells from immunized B6 mice were stimulated for 5 days with the immunizing peptide and antigen presenting cells under either Th17 (left panels) or Th1 (right panels) polarizing conditions; then the numbers of IL-17^+^ and IFN-γ^+^ cells among the TCRαβ^+^ responder T cells was assessed after intracellular staining with anti-IL-17 and anti-αβTCR mAbs. Upper panels are the responses stimulated by CD73^+/+^ splenic APCs and lower panels are responses stimulated by CD73^−/−^ splenic APCs **(C)**. ELISA assays for IL-17 and IFN-γ production by responder αβ T cells after 48 h stimulation by the immunizing antigen and CD73^+/+^ or CD73^−/−^ splenic APCs. CD73^+/+^ and CD73^−/−^ BMDCs, with or without a prior treatment of splenic APCs with adenosine monophosphate. The data are from one single experiment and are representative of those obtained in three independent experiments. ns, not significant.

To determine whether CD73 expressed on splenic DCs is also functionally important, we examined the AP function of CD73^+/+^ (isolated from B6 mouse) and CD73^−/−^ (isolated from CD73^−/−^ mouse) splenic DCs. As demonstrated in **Figure 4A**, approximately one third of the splenic CD11c^+^ cells in wt-B6, but not the CD73^−/−^ mouse are CD73^+^. αβTCR^+^ responder T cells separated from immunized B6 mice were stimulated for 5 days *in vitro* with the immunizing antigen and irradiated splenic DCs, under Th1- or Th17-polarizing conditions. Evaluating numbers of IFN-γ^+^ and IL-17^+^ T cells among responder T cells shows that AMP enhances Th17 responses (**Figure 4B**, left panels) stimulated by CD73^+/+^, but not CD73^−/−^, splenic DCs and it inhibits Th1 responses (**Figure 4B**, right panels) stimulated by CD73^+/+^, but not CD73^−/−^ as well. Cytokine test (**Figure 4C**) showed that responder T cells produced comparable amounts of IFN-γ and IL-17 when stimulated by either CD73^+/+^ or CD73^−/−^, splenic DCs in the absence of AMP. In the presence of AMP, however, increased IL-17 production was seen in stimulation of responder T cells by CD73^+/+^, but not CD73^−/−^, splenic DCs. Also, AMP inhibits IFN-γ production of responder T cells stimulated by CD73^+/+^, but not CD73^−/−^, splenic DCs.

### CD73 Expressed by αβ T Cells Does Not Contribute to the AMP Enhancing Effect

**Figure 5:**
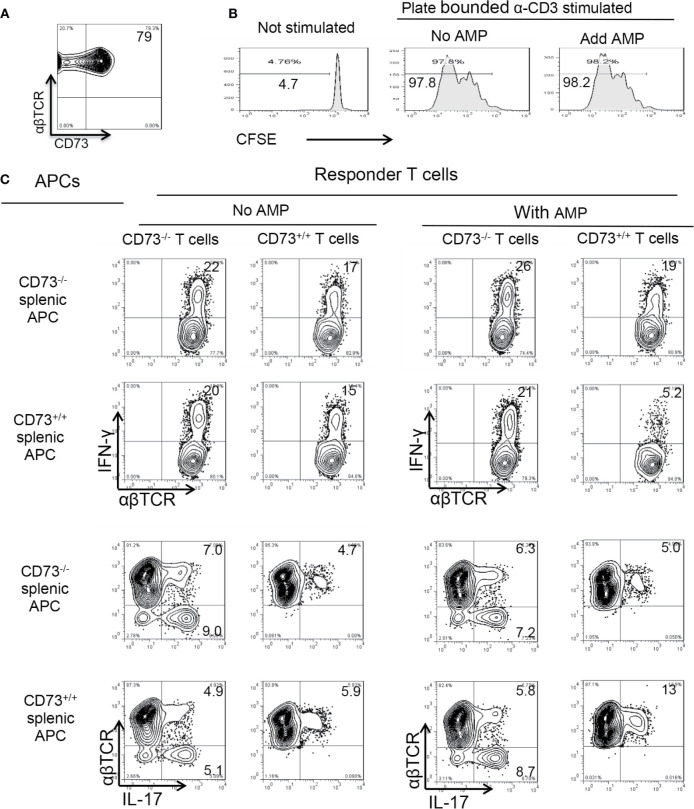
Role of CD73 on splenic dendritic cells **(A)**. CD73 is constantly expressed in >60% of αβTCR^+^ T cells. MACS column enriched αβ T cells were stained with anti-αβ TCR and anti-mouse CD73, followed by FACS analysis **(B)**. CD73 expressed on αβ T cells is not responsible for adenosine monophosphate (AMP) enhancement of Th17 responses. MACS column enriched αβ T cells were CFSE (5 μM) stained. 1.5 × 10^6^/well T cells were seeded into 24-well plate which were precoated with anti-CD3 antibodies. with or without AMP (1 µM). Some 48 h later the T cells were harvested and subjected to FACS analysis **(C)**. CD73^+/+^ splenic dendritic cells (DCs), but not CD73^+/+^ αβ T cells are responsible for AMP enhancement of Th17 responses. Crisscross test for determination of whether AMP has a direct effect on αβ responder T cells or splenic DCs. Responder αβ T cells and splenic DCs were isolated from CD73^+/+^ (B6) and CD73^−/−^ mice, respectively. The data are from one single experiment and are representative of those obtained in three independent experiments.

CD73 is constantly expressed in ~80% of αβ T cells (**Figure 5A**). To determine whether CD73 molecules expressed on αβ T cells contribute to AMP-mediated enhancement of Th17 responses, αβTCR^+^ responder T cells enriched by MACS column were CFSE labeled before stimulating with plated bound anti-CD3 antibodies, in the absence or presence of AMP (1 µM), under Th17 polarizing conditions. The results show that AMP treatment does not significantly affect the Th17 responses (**Figure 5B**), which agreed with our previous finding that CD73 expressing αβ T cells are incapable of degrading AMP to adenosine (26). To further exclude the possibility that CD73 expressed on BMDCs but not on αβ T cells accounted for the augmented Th17 responses, we conducted a crisscross test in which responder T cells isolated from immunized CD73^+/+^ (B6) and CD73^−/−^ (CD73^−/−^ mouse) mice were stimulated by CD73^+/+^ and CD73^−/−^ splenic DCs. The results showed that AMP enhancement of Th17 responses is seen only in the presence of CD73^+/+^ splenic DCs (**Figure 5C**). AMP suppressed Th1 responses, which is also seen only in the presence of CD73^+/+^, but not CD73^−/−^, splenic DCs, suggesting that CD73 expressing DCs are required in degradation of AMP to adenosine leading to inhibited Th1 and enhanced Th17 responses.

### Requirement of CD73^+/+^ BMDCs in DC γδ T Cell Interaction

**Figure 6:**
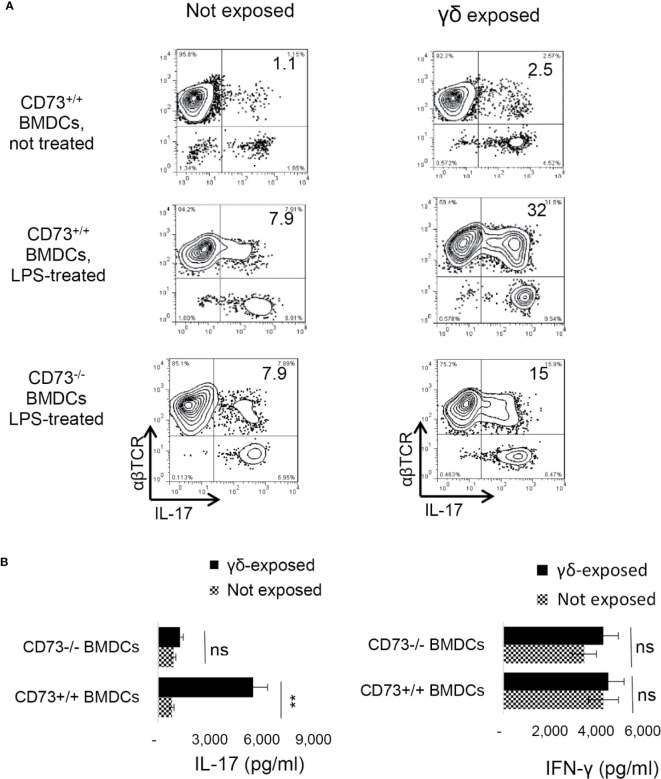
Requirement of CD73^+/+^ bone marrow dendritic cells in dendritic cell γδ T cell interaction **(A)**. Three differently prepared bone marrow dendritic cells (BMDCs) were compared for their interactive ability with γδ T cells—the BMDCs isolated from B6 mouse (CD73^+/+^), with (mid panels) or without (top panels) a prior exposure to lipopolysaccharide (LPS, 100 ng/ml)), and the BMDCs isolated from CD73^−/−^ mouse treated with LPS (lower panels). The γδ T cells were separated from immunized B6 mice using MACS column (38). After incubation with γδ T cells at a ratio of T: DC = 1:20 for 24 h. The γδ T cells were removed and the BMDCs were collected, irradiated, and seeded on to 24-well plates (5 × 10^5^/well) for AP functional test. To determine antigen presenting function, responder T cells isolated from immunized B6 mice were co-cultured with BMDCs at a ratio of T: DC = 20:1, in the presence of immunizing antigen (IRBP_1–20_) and under Th17 polarizing conditions. IFN-γ^+^ and IL-17^+^ T cells among responder T cells were examined after 5-day *in vitro* stimulation **(B)**. ELISA assays for IL-17 and IFN-γ production by responder T cells after 48 h stimulation by the immunizing antigen and CD73^+/+^ or CD73^−/−^ BMDCs. CD73^+/+^ and CD73^−/−^ BMDCs, with or without a prior exposure to γδ T cells. The data are from one single experiment and are representative of those obtained in three independent experiments. ns, not significant.

We have observed that mouse BMDCs acquired an increased ability to enhance Th17 responses after exposure to γδ T cells, and a significant portion of the BMDCs expressed CD73 after exposure to γδ T cells. To determine whether CD73 expression by BMDCs is required for DC-γδ interaction, we compared Th17 responses presented by CD73^+/+^ and CD73^−/−^ BMDCs, after exposure to γδ T cells. Three BMDC preparations were compared for their interactive ability with γδ T cells—the CD73^+/+^ BMDCs with (**Figure 6A**, mid panels) or without (**Figure 6A** top panels) prior exposure to LPS and the CD73^-/-^ BMDCs treated with LPS (**Figure 6A**, lower panels). The γδ T cells were separated from immunized B6 mice using a MACS column (38). BMDCs were exposed to γδ T cells at a pre-determined optimal ratio of T: DC = 1:20 for 24 h. Then, the γδ T cells were removed and the BMDCs were collected, irradiated, and seeded onto 24-well plates (5 × 10^5^/well), followed by co-culture with responder T cells. The AP function of BMDCs was assessed by measuring IFN-γ^+^ and IL-17^+^ T cells among responder T cells and cytokine production, under Th1-, or Th17-polarizing conditions (27, 38). Results show that the LPS-treated **(** **Figure 6A**, mid panels), but not the untreated (**Figure 6A**, Top panels), CD73^+/+^ BMDCs stimulated a significantly greater number of IL-17^+^ cells among responder T cells, after exposure to γδ T cells. However, the CD73^-/-^ BMDCs were unable to augment Th17 response by exposure to γδ T cells (**Figure 6A**, lower panels). Cytokine tests showed that the LPS-treated, but not the untreated, CD73^+/+^ BMDCs induced greater amounts of IL-17 production of responder T cells after interacting with γδ T cells (**Figure 6B**). IFN-γ production of responder T cells remained unchanged after the BMDCs were exposed to γδ T cells, further supporting the prediction that expression of CD73 by DCs crucially triggers a strong Th17 response; whereas disabling CD73 function on DCs prevents higher Th17 responses.

## Discussion

Under pathologic conditions, a large amount of ATP is released into the extracellular compartment by injured and stressed cells (3, 4). Extracellular ATP acts on many immune cells to promote inflammation (5–9). Due to the potent immune stimulatory actions of ATP, the extracellular concentrations are kept in check by enzymatic digestion of ATP. The ecto-enzyme CD73 (ecto-5’-nucleotidase), a molecule pivotally involved in converting non-immunosuppressive AMP into immunosuppressive adenosine (12, 16), is expressed by many cell types, including Treg cells (17, 21, 42, 43), B cells (44) and endothelial cells (45). Previous studies showed that myeloid cells express altered levels of CD73 depending on their activation state (46), which is closely associated with pro- and anti-inflammatory functions of myeloid cells (47–50). In addition, the function of regulatory cells relies on expression of CD39 and CD73, and generation of adenosine mediates the immunosuppressive ability of regulatory T cells (Tregs) (21).

Adenosine is an important regulatory molecule which modulates a wide range of physiological functions (51), including the immune response (51–54), by acting on T cells (16, 55), macrophages/DCs (24, 56), NK cells (57), neutrophils (58), platelets (59), and regulatory T cells (25, 60). Previous studies showed that adenosine diminishes the capacity of DCs to initiate and amplify Th1 immune responses and that CD73 expressed on DC/macrophages is anti-inflammatory. For example, M2 macrophages generate an adenosine-rich environment, which in turn can augment the anti-inflammatory and tissue remodeling activities of these cells (11, 23). Here we show that BMDCs preferentially activate Th1 responses; after treatment with TLR ligands, both Th1 and Th17 stimulating effects of BMDCs were enhanced. However, dual treatment with LPS and adenosine enabled BMDCs to acquire greatly increased Th17 promoting activity. Both Th1 and Th17 pathogenic T cells contribute to the pathogenesis of autoimmune diseases (61–63), and levels of extracellular adenosine increase greatly during inflammation (32, 33). The opposite effect of adenosine on Th1 and Th17 responses could certainly offset therapeutics targeting Th1 pathogenic reactions. As such, clarification of the conflicting effect of adenosine on Th1 and Th17 responses is of great importance.

Given the pivotal function of CD73 in adenosine-mediated immunoregulation (12, 15), and the previous finding that immune cells that express different levels of CD73 are functionally distinct (24, 25), we examined the role of CD73 on DCs and investigated whether CD73^+^ DCs are functionally exceptional in Th1 and Th17 responses. Studies have reported that only ~2–10% of freshly isolated human monocytes express CD73 (64). In mouse BMDCs, CD73 was easier to find in immature BMDCs than in mature BMDCs (65). M1 macrophages have been reported to exhibit a modest decrease in the expression of both CD39 and CD73, while M2 macrophages express higher levels of both (11). Our results showed that GM-CSF-cultured BMDCs do not constantly express CD73; however, after exposure to TLR ligand or interaction with (γδ) T cells, a significantly increased number expressed CD73 which was closely associated with an increased ability to promote Th17 responses. We were also able to show that only a portion of the splenic DCs express CD73 (**Figure 4**). Functional comparisons between CD73^+^ and CD73^−^ APCs showed that while AMP has an enhancing effect on Th17 responses *via* splenic DCs, expression of CD73 on splenic DCs is required. Such observation agrees with previous findings that the mature mouse BMDCs and mature human monocyte derived DCs are less efficient than immature DCs in supporting Th17 differentiation (66, 67). Our observation complements the previous scenario by showing that adenosine diminishes the capacity of DCs to amplify Th1 immune responses (13, 14), we additionally show however adenosine augments Th17 responses in which CD73^+^ DCs play an important role. Such an observation also supports our previous observation that adenosine tips the Th1/Th17 responses towards the latter (38, 39).

The previous conclusion that CD73 is important for the anti-inflammatory immune responses has mainly been proven in Th1 responses. To determine whether such a conclusion also applies to Th17 responses we have compared functions of CD73^+/+^ and CD73^−/−^ DCs in Th1 and Th17 responses. Our results showed that the CD73^+/+^ DCs have significantly greater Th17 enhancing effects, particularly when AMP is provided. It is likely that the CD73^+^ DCs have an increased ability to convert AMP to adenosine, leading to inhibited Th1 responses but enhanced Th17 responses. It is important to note, however, that CD73 molecules expressed on different immune cells are functionally different. We have previously reported that γδ T cells have a greater enhancing effect on Th17 responses when they express less CD73 (26, 27); in this study we show that BMDCs acquire a stronger Th17 stimulating activity when they express CD73. Given that Th17 cells uniquely express CD39/CD73 (42, 67, 68), it is likely that initiation of Th17 responses by CD73^+^ DCs may trigger cascading responses in which activated Th17 cells become CD73-providing cells in the responses which further facilitate the conversion of AMP to adenosine. Given our previous observation that CD73^+^ DCs have increased ability to activate γδ T cells and that CD73-expressing γδ T cells are much more potent in converting AMP to adenosine (26), we conclude that the induction of CD73^+^ BMDCs cells will lead to cascading Th17 responses *via* several pathways.

In summary, stimulation of adenosine receptors skews DC differentiation (69–71). In this study, we show that conversion of AMP into adenosine by CD73 expressing DCs is an important pathway in triggering cascading T17 responses.

## Data Availability Statement

The raw data supporting the conclusions of this article will be made available by the authors, without undue reservation.

## Ethics Statement

The animal study was reviewed and approved by Institutional Animal Care and Use Committee of University of California Los Angeles (Protocol number: ARC # 2014-029-21).

## Author Contributions

DS, HK, and HS designed research. DS and MK performed the experiments and analyzed data. DS and HK wrote the manuscript. All authors contributed to the article and approved the submitted version.

## Funding

This work was supported by NIH grants EY0022403 and EY018827 and grant from for Research to Prevent Blindness, NYC.

## Conflict of Interest

The authors declare that the research was conducted in the absence of any commercial or financial relationships that could be construed as a potential conflict of interest.
